# Severe psychiatric disorders are associated with increased risk of dementia

**DOI:** 10.1136/bmjment-2024-301097

**Published:** 2024-06-17

**Authors:** Joshua Stevenson-Hoare, Sophie E Legge, Emily Simmonds, Jun Han, Michael J Owen, Michael O’Donovan, George Kirov, Valentina Escott-Price

**Affiliations:** 1 Cardiff University, Cardiff, UK; 2 UK Dementia Research Institute at Cardiff University, Cardiff, UK

**Keywords:** Data Interpretation, Statistical, Schizophrenia & psychotic disorders, Depression & mood disorders, Adult psychiatry

## Abstract

**Background:**

Individuals with psychiatric disorders have an increased risk of developing dementia. Most cross-sectional studies suffer from selection bias, underdiagnosis and poor population representation, while there is only limited evidence from longitudinal studies on the role of anxiety, bipolar and psychotic disorders. Electronic health records (EHRs) permit large cohorts to be followed across the lifespan and include a wide range of diagnostic information.

**Objective:**

To assess the association between four groups of psychiatric disorders (schizophrenia, bipolar disorder/mania, depression and anxiety) with dementia in two large population-based samples with EHR.

**Methods:**

Using EHR on nearly 1 million adult individuals in Wales, and from 228 937 UK Biobank participants, we studied the relationships between schizophrenia, mania/bipolar disorder, depression, anxiety and subsequent risk of dementia.

**Findings:**

In Secure Anonymised Information Linkage, there was a steep increase in the incidence of a first diagnosis of psychiatric disorder in the years prior to the diagnosis of dementia, reaching a peak in the year prior to dementia diagnosis for all psychiatric diagnoses. Psychiatric disorders, except anxiety, were highly significantly associated with a subsequent diagnosis of dementia: HRs=2.87, 2.80, 1.63 for schizophrenia, mania/bipolar disorder and depression, respectively. A similar pattern was found in the UK Biobank (HRs=4.46, 3.65, 2.39, respectively) and anxiety was also associated with dementia (HR=1.34). Increased risk of dementia was observed for all ages at onset of psychiatric diagnoses when these were divided into 10-year bins.

**Conclusions:**

Psychiatric disorders are associated with an increased risk of subsequent dementia, with a greater risk of more severe disorders.

**Clinical implications:**

A late onset of psychiatric disorders should alert clinicians of possible incipient dementia.

**WHAT IS ALREADY KNOWN ON THIS TOPIC**

- Severe mental illness has been shown to increase the risk of dementia in later life. The effect is strongest for schizophrenia/psychosis, followed by depression with a nearly twofold increase in relative risk. However, some of the previous research has suffered from bias and the risk among people with bipolar disorder and anxiety is less well established.

**WHAT THIS STUDY ADDS**

- We compare the effect of different types of psychiatric disorders within the same large longitudinal cohorts and show that the risk of dementia correlates with the severity of the psychiatric disorder. The effect is present for a range of ages at onset but there is a steep increase for disorders that first present in the few years prior to dementia diagnosis.

**HOW THIS STUDY MIGHT AFFECT RESEARCH, PRACTICE OR POLICY**

- Clinicians should be alert to the increased risk of dementia in people with psychiatric disorders and to the possibility that a late onset could be a prodromal sign of dementia. Future research should try to find out whether better treatments of psychiatric illness can also reduce the risk of dementia.

## Background

Individuals who experience certain psychiatric disorders have been shown to have a greater risk of developing dementia than the general population.^1^ The strength of this association may have even been underestimated as the greatest risk of dementia onset is in later life and individuals with psychiatric disorders have a reduced lifespan, that is, they are more likely to die before they develop dementia.^2^ The strongest evidence is for depression, with increased risk of all-cause dementia reported in 30 of 33 studies (pooled relative risk (RR) of 1.96, 95% CI: 1.59 to 2.43).^3^ Schizophrenia is the psychiatric disorder associated with the highest risk of dementia, with risk being reported in a recent population-based study from Denmark to be increased around threefold^4^ and even higher in another large US-based medical insurance dataset.^5^ The most recent meta-analysis of longitudinal studies identified seven relevant studies which allowed an estimated increased RR of dementia following psychotic disorders (mostly schizophrenia) of 2.19 (95% CI: 1.44 to 3.31).^3^ Bipolar disorder has also been associated with increased dementia risk as well,^3 6^ but only five studies met selection criteria in the latest meta-analysis by Stafford *et al* 3 and they were based on only two population samples, from Taiwan and Western Australia. There is no consistent evidence that anxiety is associated with dementia although it is highly comorbid with most other psychiatric disorders.^3 7^

One problem in interpreting these associations is that individuals can experience psychiatric symptoms after dementia onset.^8^ It remains unclear to what extent this is a direct effect of neurodegenerative pathology or if these effects are mediated by the psychological and social consequences of dementia. Another issue is that, in some cases, psychiatric symptoms may be prodromal manifestations of the dementia process, for example, frontotemporal dementia can first present with psychotic symptoms,^9^ and it has been reported that psychiatric symptoms may predate a clinical diagnosis of dementia by up to 5 years.^10 11^

Although many studies have examined the relationships between psychiatric disorders and dementia, a recent umbrella review of the subject concluded these have produced low strength of evidence and had a high risk of bias.^12^ Thus, there was evidence of high heterogeneity due to different study designs and quality of the studies, variation of diagnosis and definition of dementia, as well as possible publication bias.^12^ Moreover, most studies reported only one or two disorders at a time, making comparisons between disorders problematic. A systematic review concluded that while the evidence for depression was very strong, there is only limited longitudinal evidence in the literature for the effect of anxiety, bipolar disorder or psychotic disorders, again raising the need for further large population-based and longitudinal studies.^3^

## Objective

We used two data sources. The first is based on the Secure Anonymised Information Linkage (SAIL) databank which contains routinely collected healthcare data (including primary care data) for approximately 80% of the population of Wales, UK.^13^ The participants are representative of the Welsh population with regard to age, sex and deprivation, and were residents in Wales between 1970 and 2019. We included the records of individuals aged between 45 and 70 years for a period of 20 years and tracked both the development of dementia and psychiatric events. We employed survival models that accounted for age, sex and deprivation (as individuals in more deprived areas might be at a higher likelihood of developing psychiatric disorders^14^). We used the same methods to analyse the data in the UK Biobank,^15^ a large cohort from the UK of individuals aged 40–70 years at the beginning of the data collection, followed up for 13–17.5 years with available primary care records.

## Methods

### SAIL databank

Diagnoses in SAIL came from the Patient Episode Database for Wales (PEDW) and the Welsh Longitudinal General Practitioner dataset (WLGP), which includes records from primary care physicians of diagnoses, treatments, symptoms and referrals. Death certificate records were not used for the diagnosis but note that 96% of those who had dementia diagnoses in their death certificates were also diagnosed in the above datasets. Demographic data on sex, age and death were sourced from the Welsh Demographic Services Database (WDSD) and from WLGP.

To assign diagnoses, we used ICD-10 (International Classification of Diseases 10th Revision) codes in PEDW and National Health Service read codes in WLGP. We grouped ICD-10 codes into four categories: schizophrenia, mania/bipolar, depression and anxiety (online supplemental table 1). For example, under ‘schizophrenia’, we included schizophrenia, schizoaffective disorder, delusional disorder, acute and transient psychotic disorders. Hospitalisation data were sourced from the PEDW dataset by querying instances of inpatient stay with a psychiatric disorder as the primary diagnosis (online supplemental table 1). For dementia, we included the most common diagnoses of dementia: Alzheimer’s disease, vascular dementia and ‘unspecified dementia’, and grouped them together as one entity (dementia).

10.1136/bmjment-2024-301097.supp1 Supplementary data

Measures of deprivation were taken from the WDSD dataset, using the Welsh Index of Multiple Deprivation 2014 statistics.^16^ Individuals’ most recent deprivation score was used to assign a decile value.

The study period was between 1 January 1999 and 31 December 2018. To ensure that individuals had an appreciable chance of developing dementia before the end of the study, and to make the age range as close as possible to that in the UK Biobank, we included only those who were aged between 45 and 70 years on 1 January 1999. Controls were individuals with no diagnoses of any of the above psychiatric disorders. Note that electronic data collection is only reliable from late 1990s and therefore earlier ages at onset are missing in this dataset. For this reason, the mean age at onset of psychiatric disorders appears to be unusually late.

### UK Biobank

The UK Biobank is a cohort of around half a million volunteers from the UK recruited between 2006 and 2010 when they were between 40 and 70 years of age. Participants were approached by mail and those who consented to take part were recruited. The study collected in-depth genetic and health information on participants, including records of hospital admissions, primary care data, a variety of self-reported information, including medical problems and cognitive tests.

Diagnoses of disorders in UK Biobank were derived using ICD-10 codes (online supplemental table 1) based on information from hospital admissions, death certificates, self-reported conditions and primary care records as provided by the UK Biobank category 1712, ‘First occurrences’. This variable provides the date of the first report of any ICD-10 codes using all the above information. To make the data comparable with SAIL, only UK Biobank participants with available primary care data were included (N=228 937). Similar to SAIL, due to electronic records starting in later years, some early-onset cases would have been wrongly given a later age at onset in the UK Biobank, although the self-report data reduce this problem. The first recorded age at onset of psychiatric diagnoses was 8–13 years earlier in the UK Biobank (table 1). The participants were recruited from March 2006 to September 2010. Our observation period ended with the last data download in September 2023, providing between 13 and 17.5 years of longitudinal observation. Diagnoses made before the recruitment point were also accepted.

**Table 1:** Demographics of individuals in SAIL and UK Biobank with schizophrenia, mania/bipolar, depression, anxiety (assigning people to a single diagnostic category corresponding to their highest-ranking diagnosis) and controls

Diagnostic group	SAIL (N=1 048 081)							UK Biobank (N=228 937)						
	N (% of the sample)	Mean deprivation	Age at onset (SD)	N died (%)	N dementia (%)	Mean age at the end of study (SD)	Age at dementia (SD)	N (% of the sample)	Mean deprivation	Age at onset (SD)	N died (%)	N dementia (%)	Mean age at the end of study (SD)	Age at dementia (SD)
Schizophrenia	10 097 (1.0)	3.9	61.9 (13.1)	5195 (51.5)	2096 (20.8)	72.1 (8.0)	72.9 (9.2)	1172 (0.5)	5.4	48.0 (16.5)	272 (23.2)	102 (8.7)	68.5 (8.0)	72.0 (5.9)
Mania/bipolar	4691 (0.4)	4.5	60.2 (12.9)	1910 (40.7)	789 (16.8)	72.3 (7.4)	72.7 (8.8)	914 (0.4)	5.6	52.3 (13.4)	159 (17.4)	67 (7.3)	70.1 (7.9)	72.5 (5.5)
Depression	110 465 (10.5)	4.4	61.0 (13.8)	34 413 (31.2)	12 803 (11.6)	73.1 (7.2)	75.3 (8.4)	32 697 (14.3)	5.9	48.0 (13.4)	3627 (11.2)	1079 (3.3)	69.9 (7.8)	73.0 (6.2)
Anxiety	31 991 (3.1)	4.8	65.3 (12.7)	9347 (29.2)	2664 (8.3)	74.1 (7.2)	76.8 (8.4)	20 328 (8.9)	6.4	52.4 (12.2)	1781 (8.8)	424 (2.1)	71.1 (7.9)	73.9 (5.9)
Controls	890 837	4.7	NA	241 156 (27.1)	42 811 (4.8)	73.7 (7.3)	76.6 (8.3)	173 826	7.4	NA	13 704 (7.8)	2752 (1.6)	71.2 (8.0)	74.9 (5.9)

The mean age at the end of follow-up is based on the ages of those who were still alive, or the age at death, or the age at dementia diagnosis, whichever came first. The mean deprivation is calculated as the average over the derivation index’s decile membership with first decile representing the most deprived areas.

NA, not available; SAIL, Secure Anonymised Information Linkage.

### Data analysis

In both datasets (SAIL and UK Biobank), a hierarchical diagnostic group membership was generated, and individuals were assigned to a single diagnostic category corresponding to their highest-ranking diagnosis in terms of severity. Membership was assigned in the following order: (1) schizophrenia, (2) mania/bipolar, (3) depression, (4) anxiety. For example, people with ‘depression’ had not ever received a diagnosis of ‘schizophrenia’ or ‘mania/bipolar’. Individuals with none of these diagnoses by the end of the observation period were assigned as controls. This hierarchical approach for diagnoses was used for all analyses. In addition, we excluded people with psychiatric disorder who were first diagnosed within a year of dementia diagnosis or any time after it.

Cox regression survival models were employed to examine the relationships between psychiatric disorders and dementia. Dementia data were censored at age at diagnosis of dementia, or, in those who did not develop dementia, either the age at the end of the study period or at death, when it occurred earlier. Dementia risk was estimated using diagnostic group membership, controlling for sex and deprivation. We also investigated the risk of dementia in groups of individuals divided in 10-year bins (<40, 40–49, 50–59, 60–59, 70+) according to their age at onset of first diagnosis of the respective psychiatric disorder. Each separate bin was compared against the full set of controls.

The p values are reported without correction for multiple testing but, where appropriate, we state whether the p values would survive correction for multiple testing. The Bonferroni-corrected significance threshold was set to 0.001[=0.05/48]: association with dementia in the whole sample and in five age subgroups according to the age at onset of psychiatric disorder (<40, 40–49, 50–59, 60–69, 70+) for four psychiatric conditions in two databanks (6×4×2).

## Findings

After removal of people who did not meet the inclusion criteria, the SAIL databank consisted of 1 048 081 individuals (49.9% males). An overview of the demographics and recorded first age of diagnosis of psychiatric disorders of included individuals is shown in table 1. The age distribution of participants in the two datasets was markedly different due to the different structure of the databanks (online supplemental figure 1). By the end of the study period, 61 163 people had developed dementia (5.8% of the sample) and 292 021 people had died (27.9%). The hierarchical categorisation method resulted in 10 097 individuals classified with schizophrenia, 4691 with mania/bipolar, 110 465 with depression and 31 991 with anxiety. There were 890 837 individuals without any of these psychiatric diagnoses, used as controls.

Of the 228 937 UK Biobank participants included in this study, 4424 had been diagnosed with dementia by 2023 (1.9% of the sample) and 19 543 people had died (8.5% of the sample). The numbers of people with schizophrenia, mania/bipolar, depression and anxiety after hierarchical categorisation were 1172, 914, 32 697 and 20 328, respectively. There were 173 826 individuals without any of these psychiatric disorders (controls).

The proportion of people with psychiatric diagnoses who had died by the end of the follow-up period was much higher than in controls, in both datasets, especially for the schizophrenia and mania/bipolar groups but nearly the same for anxiety (table 1).

Mean age at dementia diagnosis in the UK Biobank (74.2, SD=6.2) was younger than in SAIL (76.1, SD=8.4), and rates of dementia (1.9%) and death (8.5%) were about three times lower than in SAIL (5.8% and 29.9%, respectively), which is expected due to the lower mean age of participants in the UK Biobank and the known selected participation bias for healthier participants in that sample.^17^

In both datasets, people from more severe psychiatric diagnosis groups had lower deprivation index. The UK Biobank participants were substantially less deprived than people from the Welsh population (SAIL) across all diagnostic groups (table 1).

**Figure 1:**
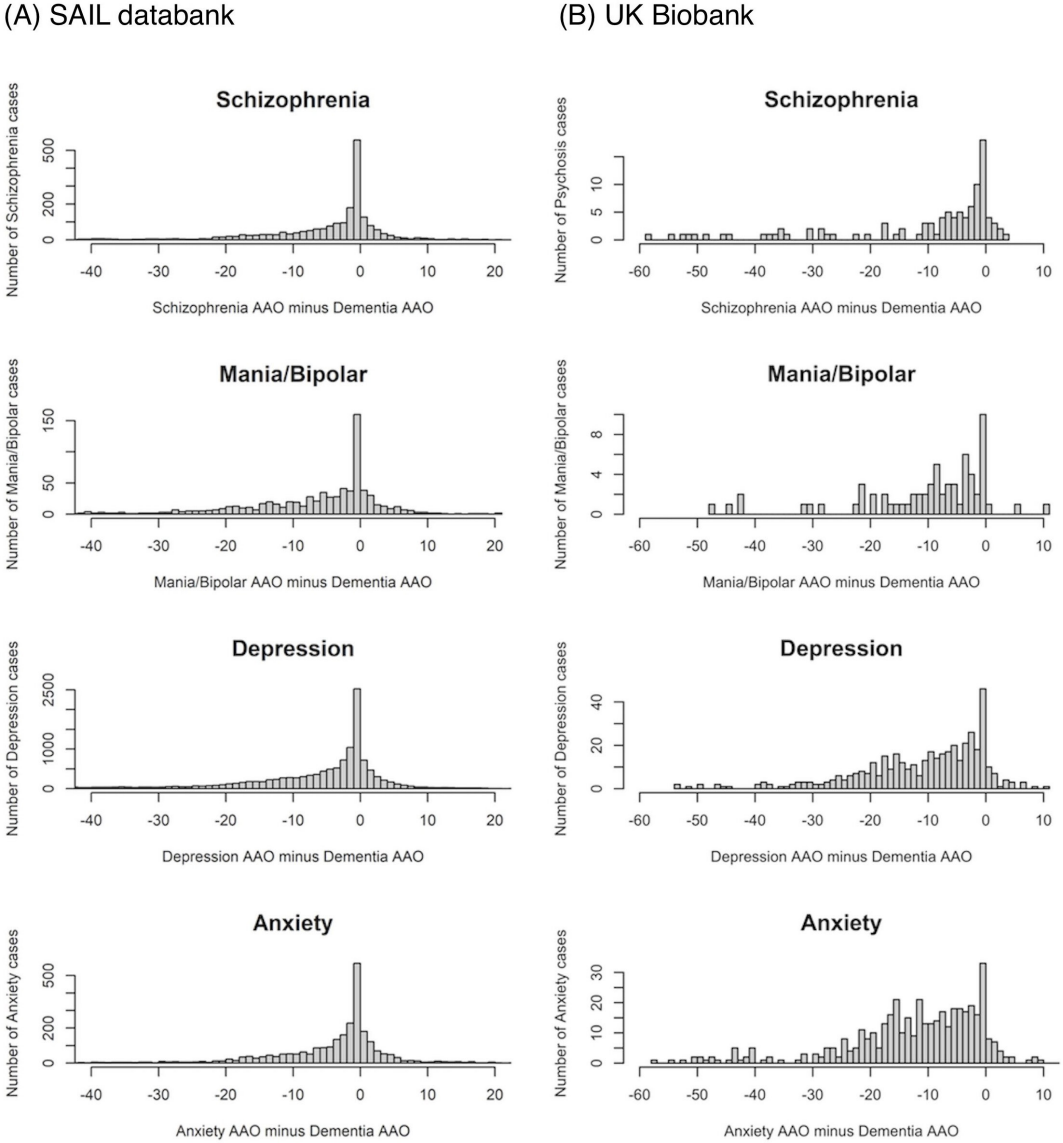
Distribution of differences between the age of first diagnosis of a psychiatric disorder and age of diagnosis for dementia in (A) Secure Anonymised Information Linkage databank and (B) UK Biobank, assigning people to the diagnostic category corresponding to their highest-ranking diagnosis. AAO, age at onset.

In both samples, the incidence of psychiatric disorder diagnoses among those who developed dementia increased towards the point of dementia diagnosis and was highest during the year preceding the dementia diagnosis for all psychiatric categories (figure 1).

### Psychiatric disorders and dementia

Figure 1 shows the distribution of the time between age at first diagnosis of the psychiatric diagnosis and age at dementia diagnosis. The most common time for psychiatric disorders to be diagnosed was within 1 year prior to dementia diagnosis. As can be seen in figure 1, there are many cases with first onset of psychiatric disorder after the onset of dementia; these were excluded from analysis. In addition, we excluded people with psychiatric disorder diagnosed first within a year prior to dementia diagnosis, as the psychiatric symptoms are likely prodromal of dementia, rather than increasing the risk of it. However, it is not clear how many years prior to dementia one should start excluding cases, as there is no clear cut-off point for risk increase. An exclusion of too many cases with both dementia and psychiatric diagnoses would create a serious bias, which might even lead to a spurious negative association between psychiatric illness and dementia. To avoid this, we split the sample with psychiatric diagnosis into 10-year bins of age at onset of psychiatric diagnosis and only excluded cases diagnosed within 1 year of dementia diagnosis (see the Methods section). This way, we were able to assess the risk of dementia when the psychiatric illness started decades earlier.

**Figure 2:**
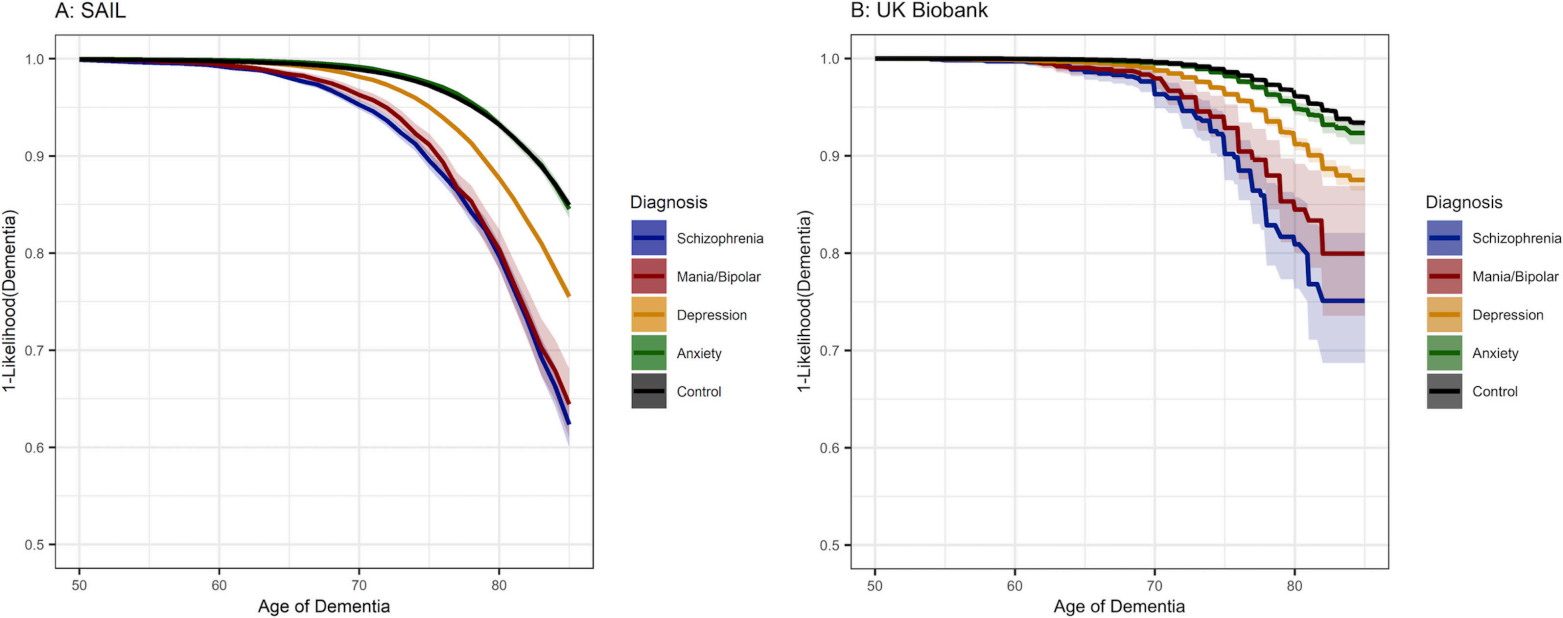
Kaplan-Meier curves for survival models in Secure Anonymised Information Linkage (A) and UK Biobank (B) estimating dementia risk by hierarchical psychiatric diagnosis group membership, controlling for sex and deprivation. Ribbons are 95% CIs.

In SAIL, after excluding from further analyses, individuals who had at least one psychiatric disorder in the year before, or anytime after the diagnosis of dementia, and using Cox regression analysis (see the Methods section), psychiatric disorders were associated with significantly increased risk of dementia (figure 2A): schizophrenia (HR=2.87 (95% CI: 2.7 to 3.04), p=9.0×10^−268^), mania/bipolar (HR=2.80 (95% CI: 2.6 to 3.1), p=8.8×10^−112^) and depression (HR=1.63 (95% CI: 1.6 to 1.7), p<1.0×10^−300^), whereas anxiety was not significantly associated (HR=0.94 (95% CI: 0.9 to 1.0), p=0.014, ns after correction for multiple testing).

In the UK Biobank, after excluding individuals who had a first psychiatric diagnosis in the year before, or anytime after the diagnosis of dementia (figure 2), schizophrenia (HR=4.46 (95% CI: 3.5 to 5.6), p=6.3×10^−37^), mania/bipolar (HR=3.65 (95% CI: 2.8 to 4.8), p=3.4×10^−21^), depression (HR=2.39 (95% CI: 2.2 to 2.6), p=3.8×10^−124^) and anxiety (HR=1.34 (95% CI: 1.2 to 1.5), p=1.3×10^−7^) were all significantly associated with an increased risk of dementia. For schizophrenia, mania/bipolar and depression, this pattern of results is the same as in SAIL, although HRs are higher. However, unlike in SAIL, anxiety was also associated with an increased risk of dementia.

Our analyses were performed after hierarchical exclusion of individuals with highest-ranking diagnosis in terms of severity of psychiatric disorder from the ‘less severe’ groups. The results without this filtering show higher and more significant HRs for mania/bipolar, depression and anxiety. This is due to the introduction of people with more severe comorbid diagnoses to these diagnostic groups and the increased sample size (data not shown).

From figure 1, it is evident that there is a gradual increase in the number of first-onset psychiatric disorders for several years prior to dementia diagnosis, with a steep rise in the final year. This raised the question whether most of the increased risk is due to late-onset psychiatric disorders. We analysed groups of individuals divided into 10-year bins according to their age at onset of psychiatric diagnosis against the full set of controls (online supplemental table 2). As above, the individuals with age at onset within a year of dementia diagnosis or after it were excluded.

**Figure 3:**
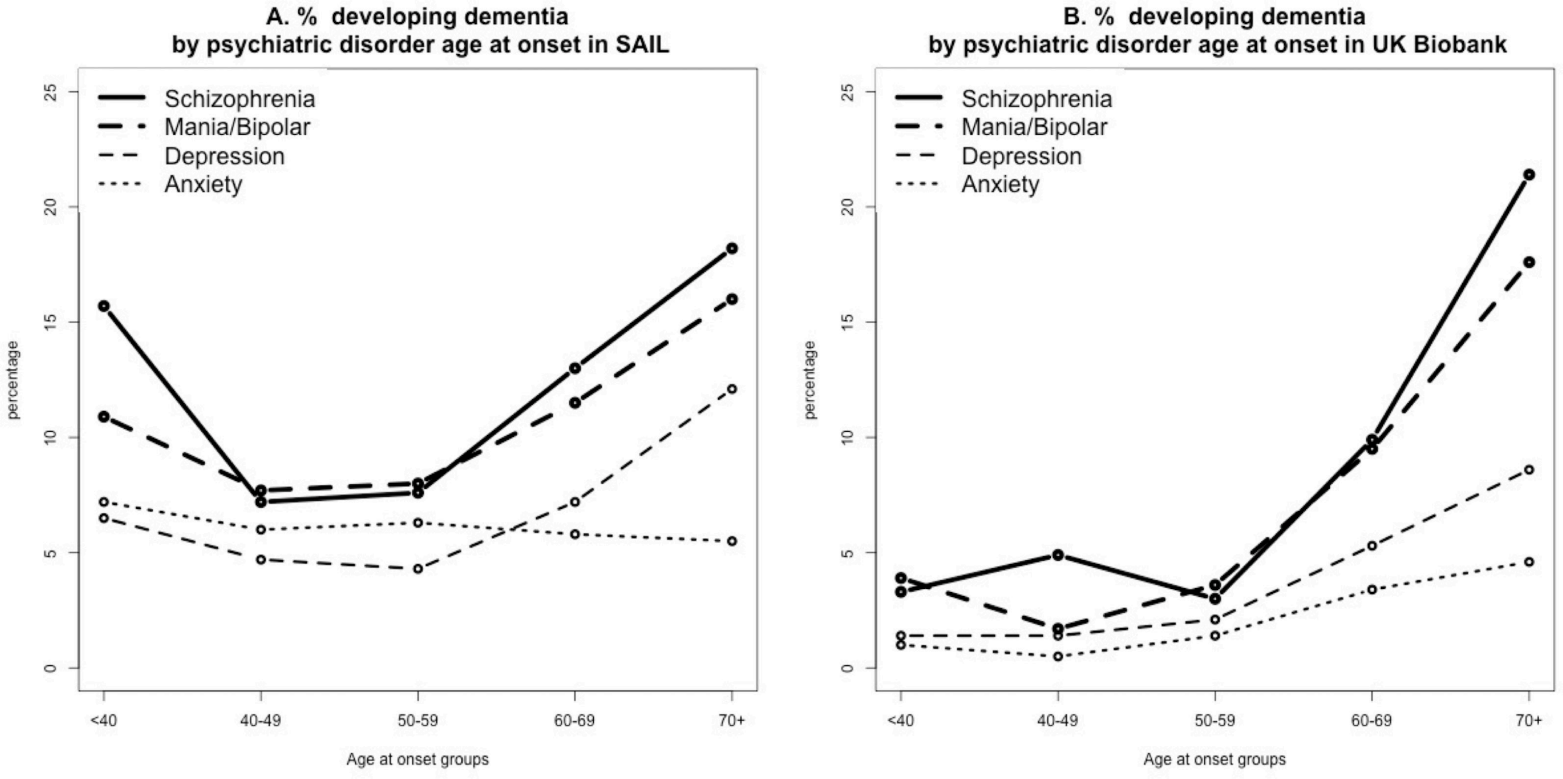
Percentage of people developing dementia corresponding to psychiatric disorder age at onset bins in (A) SAIL databank and (B) UK Biobank. Data are presented for individuals excluding those who developed psychiatric conditions in the same year or after dementia diagnosis. SAIL, Secure Anonymised Information Linkage.

In both datasets, a higher risk of dementia was associated with more severe psychiatric conditions in our hierarchical structure (figure 3 and online supplemental table 2). It should be noted that the numbers of individuals with psychiatric diagnoses and dementia in some of the age bins were small in the UK Biobank (online supplemental table 2) resulting in some large fluctuations in the lines in figure 3. However, the pattern of results is similar, with the more severe diagnoses having in general higher HRs for dementia in both datasets.

## Discussion

We used a large longitudinal population-based representative medical health record dataset from Wales, UK (SAIL) and a large cohort from the general population in the UK (the UK Biobank) to examine the relationship between psychiatric disorders and the risk of developing dementia. Previous existing literature demonstrates that differences in study design (population or case/control) and disorder ascertainment (objective health records vs volunteers) may lead to different results. The main value of our study is that we analyse two large longitudinal datasets with two different designs, comparing diagnoses in the same datasets and using the same methods.

We found that schizophrenia and related psychotic disorders, as well as bipolar disorder, are associated with a threefold to fourfold increased risk of dementia, similar to other studies reporting associations of twofold to threefold increased risk of dementia.^3 4 18^ Depressive disorders in our study were associated with increased risk of dementia (~1.6-fold to 2-fold), very similar to the 1.85-fold and 1.96-fold increased risk reported in systematic reviews.^3 19^ The small differences are likely due to different study designs, inclusion criteria and the fact that we excluded cases whose first diagnosis was within 1 year prior to, or anytime after, a dementia diagnosis.

We show that the incidence of psychiatric disorders in late life peaks just before dementia diagnosis. The peak incidence of psychiatric disorders in the year prior to dementia diagnosis suggests that psychiatric symptomatology in many late-onset cases represents prodromal symptoms of dementia, rather than psychiatric disorders increasing risk of dementia. However, dementia and the associated psychiatric disorders may share latent risk factors, leading to their comorbid presentation in some people when an individual reaches the threshold for disease onset.

Our results show that the first onset of psychiatric disorders is increased not only during the year preceding the dementia diagnosis, but the incidence only gradually declines as a function of the number of years prior to the dementia diagnosis, without any obvious cut-off (figure 1). This raises the question whether even earlier onset of psychiatric disorders might reflect prodromal effects of dementia, similar to cognitive problems that can be detected for several years prior to dementia.^20^ To address this possibility, we analysed the data in 10-year wide age at onset bins: onset under 40 years, between 40 and 49, etc. If the association between psychiatric disorders and dementia is attributable to prodromal symptoms of dementia, we would expect the association between psychiatric disorders and dementia to be strongest for an onset of psychiatric disorder in the age ranges most typical of people with dementia. However, this is not what our analyses show; indeed in SAIL, the larger dataset, the results (online supplemental table 2) are the opposite of that expectation. Moreover, although the two datasets have a different pattern of risk increase across age groups, in both datasets, those with ages at onset of psychiatric disorders before 40 show robust evidence for association with subsequent dementia. The differences between the two datasets could be due to the much higher rates of dementia in SAIL, and to the very different age distribution of patients. However, the risk is increased in both datasets for all ages at onset, with a similar pattern for the different diagnoses. This finding could be explained by the fact that individuals who develop serious psychiatric disorders have higher than average exposure to environmental factors that increase risk of dementia, for example, smoking, recreational drug usage, poor nutrition, low levels of exercise and social isolation.^21^ Alternatively, this could reflect the fact that long-term experience of psychiatric disorders may be accompanied by neurological changes^22–24^ that increase the likelihood of dementia. Such changes could be part of or be downstream consequences of the underlying pathophysiology of the psychiatric disorders per se or its treatment with psychotropic medication.^21^ As our study is observational, we cannot distinguish between these two possibilities.

Our next observation is that more severe psychiatric disorders are associated with stronger risk. In both datasets, schizophrenia and related psychotic disorders exhibit the highest risk, followed by bipolar disorder and then by depression. These results are consistent for different ages at onset (with some fluctuation in the UK Biobank that may be due to very small number of cases with schizophrenia and mania/bipolar who developed dementia for some of the bins) (online supplemental table 2). Anxiety disorders show a very small increased risk in the UK Biobank and no risk in the SAIL population. The difference could reflect a nearly threefold higher rate of anxiety in the UK Biobank likely reflecting different ascertainment and diagnostic methodology, including self-report and participation bias. This might also explain the seemingly steeper increase in the incidence of a first diagnosis of anxiety in the years prior to dementia diagnosis in SAIL as compared with the UK Biobank (figure 1).

Previous work has been conflicting regarding the relationship between anxiety and dementia. As we used a hierarchical categorisation method, we were able to look at anxiety independent from the effects of other psychiatric disorders with which it is highly comorbid.^7^ It may be that the results of other studies are mixed because they have individuals with other psychiatric disorders with a stronger relationship to dementia, or with insufficient statistical power in anxiety-only cohorts. In the SAIL dataset, when all other psychiatric disorders are accounted for, anxiety showed no association with dementia (figure 2), whereas in the UK Biobank, we observed a small but significant association.

Our study has several limitations. First, the datasets we used included data spanning 20 years, during which time diagnostic criteria have changed for some disorders.^25^ Second, since the electronic health records (EHRs) are reliable from late 1990s, these records have not covered the earlier ages at onset of psychiatric disorders in both SAIL and UK Biobank datasets. Third, when comparing the results of SAIL and UK Biobank, the differences between the two datasets in the results may be due to available diagnosis codes, and differences in sample makeup, such as age distribution and participant selection bias, as pointed out earlier. Fourth, records in the UK Biobank were based additionally on self-report, which can predate the onset of EHR and thus lead to a higher rate of disorders and an earlier age at onset in the UK Biobank (see table 1). Fifth, the selection criteria we used in SAIL (age 45–70 years at the start) in order to ensure that individuals were at an appreciable liability for dementia by the end of the study and approximately match the UK Biobank age span, and the recruitment criteria for the UK Biobank (age over 40 years) resulted in a systematic bias in the recorded first age of diagnosis of psychiatric disorders towards a later onset. Furthermore, the EHRs for UK Biobank and SAIL were linked from different starting points: 1997 (England), 1981 (Scotland) and only from 1999 for Wales, giving a limited number of years of records prior to the start of our observation period, especially for SAIL (for Wales). As a result, the age at first diagnoses in both datasets was higher than typically reported in the literature.^26^ Finally, a lot of dementia remains undiagnosed and therefore not present in routine healthcare data. It might be more likely detected in people who are better known to clinical services, and this might account for an apparently higher risk in the mental disorder groups. Despite the limitations and distinct differences of the datasets (first is a general population of middle age and elderly, and second is a study of middle-aged volunteers), the similarity in findings between the two datasets is remarkable.

## Clinical implications

Serious psychiatric disorders are associated with a highly significant increase in dementia risk in both the short term (immediately prior to dementia onset) and in the long term (decades after the first onset of psychiatric disorders). These may represent two distinct forms of risk which should be considered separately by both researchers and clinical practitioners. A very late onset of psychiatric disorder, especially schizophrenia or bipolar disorder, indicates a high risk of subsequent diagnosis of dementia.

## Data Availability

Data sharing not applicable as no datasets generated and/or analysed for this study. Data may be obtained from a third party and are not publicly available. All data relevant to the study are included in the article or uploaded as supplemental information. No datasets were generated during the current study. Raw data are available following applications to the SAIL databank and to the UK Biobank.
