# Supplementary material for: Severe psychiatric disorders are associated with increased risk of dementia
Source: BMJ Ment Health. 2024 Jun 17;27(1):e301097. doi: 10.1136/bmjment-2024-301097 (PMC11184176; doi:10.1136/bmjment-2024-301097)

Supplemental Table 1. Codes used for deriving diagnoses of disorders in SAIL (ICD-10 and NHS) and UK Biobank (ICD-10).

Diagnostic group	Diagnosis	ICD-10 code(s)	NHS read ICD-10 code(s)
Dementia	Alzheimer's disease	F00, G30	Eu00, F110
	Vascular dementia	F01	Eu01
	Unspecified dementia	F03	Eu02z
Schizophrenia	Schizophrenia	F20	Eu20
	Delusional disorder	F22	Eu22
	Acute and transient psychotic disorders	F23	Eu23
	Schizoaffective disorder	F25	Eu25
Mania/Bipolar	Mania	F30	Eu30
	Bipolar disorder	F31	Eu31
Depression	Single depressive episode	F32	Eu32
	Recurrent depressive episode	F33	Eu33
	Persistent mood affective disorders	F34	Eu34
Anxiety	Phobic anxiety disorders	F40	Eu40
	Generalised anxiety disorder	F41	Eu41

Supplemental Table 2. Age of dementia diagnosis, numbers (percentages) of people developing dementia in bins corresponding to age at onset of psychiatric disorder. Hazard ratios with 95% confidence intervals from Cox regression survival models reflect risk of dementia. Data are presented for individuals excluding those who developed psychiatric conditions in the year prior to or after dementia diagnosis.

Diagnosis	Age at onset range	SAIL				UK Biobank			
		Mean age at end of follow-up (SD)	N dementia/ N total (%)	Hazard Ratio (95%CI)	p-value	Mean age at end of follow-up (SD)	N dementia/ N total (%)	Hazard Ratio (95%CI)	p-value
Schizophrenia	<40 years	70.8 (7.4)	85/542 (15.7%)	4.7 (3.8-5.9)	$1.9x10^{-46}$	66.1 (8.0)	12/362 (3.3%)	3.6 (2.0-6.3)	$1.2x10^{-5}$
	40-49	66.0 (7.2)	73/1011 (7.2%)	4.9 (3.9-6.1)	$1.5x10^{-41}$	64.5 (7.4)	12/244 (4.9%)	8.5 (4.8-15.0)	$1.5x10^{-13}$
	50-59	67.9 (6.6)	175/2316 (7.6%)	4.2 (3.6-4.9)	$4.7x10^{-80}$	67.5 (7.0)	7/233 (3.0%)	3.6 (1.7-7.6)	0.001
	60-69	72.3 (6.0)	391/3001 (13.0%)	3.6 (3.3-4.0)	$1.2x10^{-141}$	73.0 (5.0)	19/192 (9.9%)	6.3 (4.0-9.9)	$1.5x10^{-15}$
	70+	79.2 (5.1)	413/2268 (18.2%)	1.9 (1.7-2.1)	$4.2x10^{-40}$	78.4 (3.6)	24/112 (21.4%)	6.0 (4.0-9.0)	$1.8x10^{-18}$
	**All ages**	**72.1 (7.7)**	**1137/9138 (12.4%)**	**2.9 (2.7-3.0)**	$\mathbf{9.0x10^{-268}}$	**70.4 (7.9)**	**74/1143 (6.5%)**	**4.5 (3.5-5.6)**	$\mathbf{6.3x10^{-37}}$
Mania/Bipolar disorder	<40 years	70.6 (6.7)	36/331 (10.9%)	4.1 (3.0-5.7)	$2.1x10^{-17}$	65.9 (8.7)	6/152 (3.9%)	4.3 (2.0-9.7)	$3.2x10^{-4}$
	40-49	68.4 (7.2)	40/520 (7.7%)	3.7 (2.7-5.1)	$7.4x10^{-17}$	65.9 (7.7)	3/175 (1.7%)	2.4 (0.8-7.5)	n.s.
	50-59	69.4 (6.2)	93/1166 (8.0%)	3.8 (3.1-4.7)	$8.2x10^{-38}$	69.1 (6.8)	10/277 (3.6)	3.6 (1.9-6.6)	$6.2x10^{-5}$
	60-69	72.4 (5.9)	168/1462 (11.5%)	3.4 (2.9-4.0)	$2.1x10^{-56}$	74.5 (4.7)	20/211 (9.5%)	5.0 (3.2-7.8)	$5.5.x10^{-13}$
	70+	79.3 (4.9)	145/904 (16.0%)	1.8 (1.6-2.2)	$2.3x10^{-13}$	78.5 (3.6)	15/85 (17.6%)	5.1 (3.1-8.5)	$2.8x10^{-10}$
	**All ages**	**72.4 (7.1)**	**482/4383 (11.0%)**	**2.8 (2.6-3.1)**	$\mathbf{8.8x10^{-112}}$	**69.9 (7.8)**	**54/900 (6.0%)**	**3.7 (2.8-4.8)**	$\mathbf{3.4x10^{-21}}$
Depression	<40 years	71.8 (6.3)	540/8250 (6.5%)	2.0 (1.8-2.1)	$6.2x10^{-54}$	67.0 (8.3)	119/8395 (1.4%)	1.6 (1.4-2.0)	$2.7x10^{-7}$
	40-49	69.2 (6.7)	527/11322 (4.7%)	1.9 (1.7-2.1)	$2.8x10^{-48}$	66.5 (7.1)	116/8244 (1.4%)	2.2 (1.8-2.7)	$7.2x10^{-17}$
	50-59	69.5 (5.8)	1174/26997 (4.3%)	2.1 (2.0-2.3)	$6.6x10^{-142}$	71.0 (6.6)	186/8963 (2.1%)	1.7 (1.5-2.0)	$1.7x10^{-12}$
	60-69	72.2 (5.8)	2247/31303 (7.2%)	2.2 (2.1-2.3)	$4.5x10^{-285}$	75.2 (5.1)	258/4894 (5.3%)	2.4 (2.2-2.8)	$1.7x10^{-42}$
	70+	79.7 (5.1)	3355/27628 (12.1%)	1.2 (1.2-1.3)	$1.7x10^{-33}$	78.9 (3.4)	167/1951 (8.6%)	2.4 (2.1-2.8)	$2.4x10^{-28}$
	**All ages**	**73.1 (7.1)**	**7843/105500 (7.4%)**	**1.6 (1.6-1.7)**	$\mathbf{<10^{-300}}$	**70.1 (7.9)**	**846/32447 (2.6%)**	**2.4 (2.2-2.6)**	$\mathbf{3.8x10^{-124}}$
Anxiety	<40 years	73.5 (7.3)	97/1339 (7.2%)	1.3 (1.0-1.7)	0.027	65.2 (8.3)	28/2702 (1.0%)	1.4 (1.0-2.1)	n.s.
	40-49	74.1 (7.2)	115/1911 (6.0%)	1.5 (1.2-1.9)	0.0003	65.4 (7.1)	23/5082 (0.5%)	0.9 (0.6-1.3)	n.s.
	50-59	73.6 (7.3)	352/5602 (6.3%)	1.4 (1.2-1.7)	$1.6x10^{-5}$	70.8 (6.4)	97/6755 (1.4%)	1.3 (1.0-1.6)	0.016
	60-69	73.6 (7.3)	592/10232 (5.8%)	1.4 (1.2-1.5)	$1.9x10^{-9}$	75.5 (4.8)	137/4035 (3.4%)	1.6 (1.3-1.9)	$2.4x10^{-7}$
	70+	73.5 (7.3)	647/11715 (5.5%)	0.7 (0.7-0.8)	$2.4x10^{-18}$	79.5 (3.3)	78/1692 (4.6%)	1.2 (1.0-1.6)	n.s.
	**All ages**	**73.6 (7.3)**	**1803/30799 (5.9%)**	**0.9 (0.9-1.0)**	**0.0135**	**68.4 (8.1)**	**363/20266 (1.8%)**	**1.3 (1.2-1.5)**	$\mathbf{1.3x10^{-7}}$
Controls		73.7 (7.3)	42811/890,837 (4.8%)	-	-	71.2 (8.0)	2752/173,826 (1.6%)	-	-

Supplemental Figure 1. Age distribution in (A) SAIL databank and (B) UK Biobank

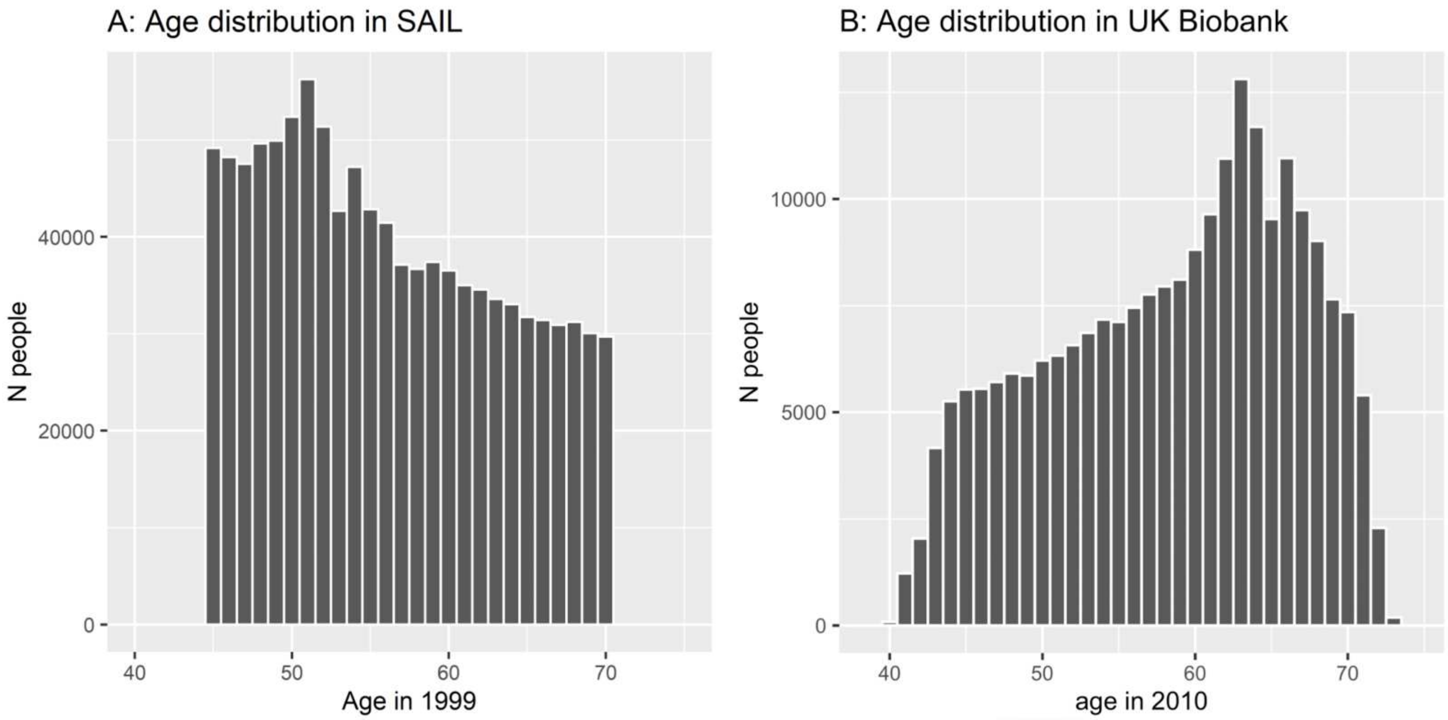

Supplement: Supplementary data [file bmjment-2024-301097supp001.pdf]
